# Improvement in the physical and mechanical properties of the cement-based composite with the addition of nanostructured BN–Fe_3_O_4_ reinforcement

**DOI:** 10.1038/s41598-021-98800-4

**Published:** 2021-09-29

**Authors:** Siavash Imanian Ghazanlou, Siamak Imanian Ghazanlou, Warda Ashraf

**Affiliations:** 1 Faculty of Materials Engineering, Sahand University of Technology, P.O. Box 51335-1996, Tabriz, Iran; 2 Nanotechnology Department, School of Advanced Technologies, Iran University of Science and Technology (IUST), 16846-13114 Narmak, Tehran, Iran; 3 Department of Civil Engineering, Center for Advanced Construction Materials (CACM), University of Texas at Arlington, Nedderman Hall, Arlington, TX 76010 USA

**Keywords:** Engineering, Materials science, Nanoscience and technology

## Abstract

In this work, the performance of modified cement by nanostructures consisting of boron nitride (BN) and iron oxide inorganic nanoparticles (Fe_3_O_4_) was analyzed. The mechanical strength, electrical resistivity, and the degree of cement hydration as well as the microstructure were investigated in detail. A hybrid filler boron nitride-iron oxide (BN–F) composed of Fe_3_O_4_ and BN was successfully synthesized using a chemical reaction. Transmission electron microscope (TEM) results showed proper binding of BN–F nanostructures. Addition of the hybrid nanostructured BN–F5 (containing 0.5 wt.% Fe_3_O_4_ and 0.5 wt.% BN) into the cement matrix increased the compressive strength and flexural strength by 65%, and 74%, respectively, after 28 days of curing. The improvement in mechanical strength is attributed to the increased surface friction induced by the Fe_3_O_4_ nanoparticles on the BN surfaces, resulting in increased interaction with the matrix. Microstructural studies, such as scanning electron microscope (SEM), showed the formation of a dense structure due to improved dispersion in the cement environment and hybrid performance in preventing crack growth, which is the main reason for the overall improvement in mechanical properties. The concrete resistance gauge (RCON, Giatec) and simultaneous thermal analysis (STA) tests revealed a significant increase in thermal and electrical conductivity in composite reinforced with nanostructured BN–F.

## Introduction

Concrete, the most consumed material in the world, exceeds the per capita output of any other material with an estimated annual consumption of 30 billion tonnes and growing global demand^1,2^. The environmental outcomes of concrete manufacturing can be local, regional, or global in scale^3,4^. Therefore, it is clear that small changes in this area can have significant consequences for controlling the environmental burden of this product^2^.

In many investigations, the effects of nanoparticle reinforcements on the improvement of the performance of cementitious materials have been mentioned. Some papers have shown that the addition of boron nitride nanoparticles can lead to a significant increase in the physical and mechanical properties of the composite^5^. Also, graphene oxide (GO) nanoplates can effectively improve the thermal conductivity of cement^6,7^. Cement composites have low thermal and electrical conductivity. Therefore, the development of high electrical and thermal conductivity cement composites is of specific importance for various applications^8^, including self-sensing composites^9^.

The layered structure of hexagonal boron nitride (H-BN) is an inapt filler material to enhance the mechanical strength of the composites due to the highly anisotropic mechanical strength^10^. However, recent developments in H-BN exfoliation provide new opportunities to use them as nanofiller material^11,12^. Boron nitride nanosheets or “white grapheme” consist of a few layers of hexagonal boron nitride planes and have several unique properties compared to graphene nanoplates such as a broad bandgap, high resistance to oxidation, high thermal conductivity and stability, electrical-insulating properties, and well chemical inertness^13–17^.

Nanofillers in the form of zero dimensions (0D, i.e. Fe_3_O_4_, nanoparticle), 1D (i.e. CNT), and, most recently, 2D (i.e. graphene, boron nitride) can strengthen cement matrix^18^. This improvement capacity originates from the outstanding mechanical properties of the nanomaterials, due to the natural strength of nanomaterials, as well as the deterrence of crack propagation. Accordingly, the key agents that affect the final properties of cement nanocomposites are the chemical composition, the geometry of additives (shape and size), and the adaptability between the nanofiller and the cement matrix. The adaptability, generally attained from chemical modification, determines the dispersion and interface interaction of main phases. Effective methods for manipulating the chemical and physical properties of nanoparticles to improve the dispersion, compatibility, and interfacial interaction of 2D nanofillers in a composite matrix are chemical modifications such as covalent and non-covalent functionalization^19,20^.

The BN sheet-shaped structure presents a challenge for the construction of high-performance uniform composites. First of all, it is difficult to achieve homogeneous dispersion of BN and Fe_3_O_4_ in a cement matrix^21,22^. Another drawback is that the process of bonding nanoparticles to each other can be complex. Moreover, the use of surfactant can decrease the desired properties of the prepared composite. In the present work, to solve these problems, firstly the dispersion problem was solved by connecting Fe_3_O_4_ nanoparticles to BN. Secondly, the nanoparticles were bonded to each other by a simple process with only the addition of HNO_3_. Finally, no surfactants were used in the cement environment due to the positive charges on the surface of Fe_3_O_4_ nanoparticles that helped to improve the dispersion of BN–F nanostructures.

## Methods

The cement used was Ordinary Portland cement (Type 2) with the requirements of ASTM C150 (density: 2.8 g/cm^3^). The magnetite nanoparticles of 20–40 nm (US-Nano Co., USA, purity: 99%, density: 5.22 g/cm^3^), nitric acid (Dr. Mojallali Co., I.R. Iran), and boron nitride powder (Sigma-Aldrich Co., USA, purity: 99%, density: 2.1 g/cm^3^) were purchased in this study.

**Figure 1:**
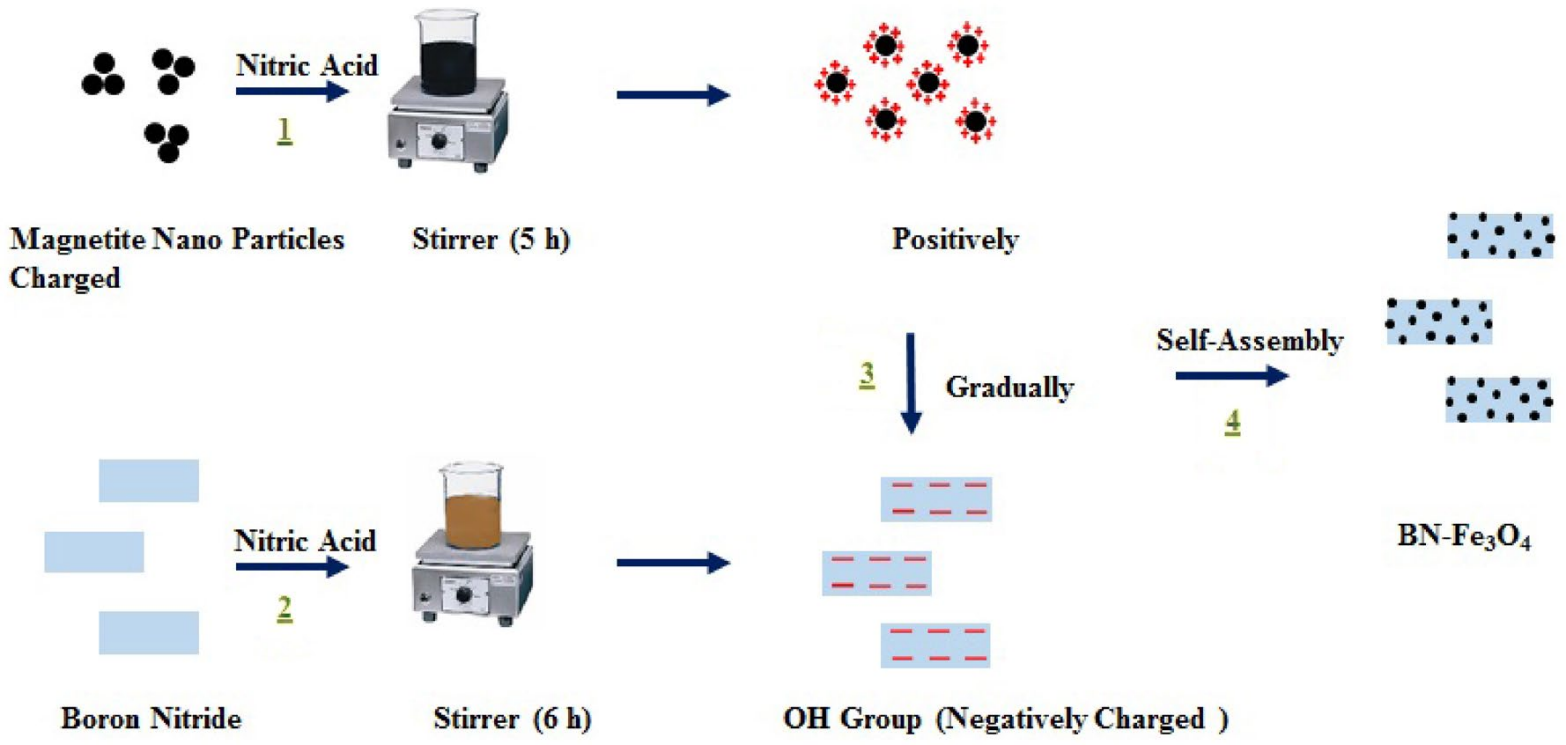
The schematic process of assembly of nanostructured BN–Fe_3_O_4_.

As can be seen in Fig. 1, firstly, a certain amount of boron nitride powder was added into the nitric acid solution, and the ultrasonic waves were applied to it for 6 h. Then, a certain amount of Fe_3_O_4_ nanopowder was sonicated in deionized water for 20 min. Next, a desirable amount of 68% nitric acid was dropwise added to the magnetite suspension during stirring for 5 h. The acidic solutions were then washed 5 times using deionized water to remove additional acid. A certain amount of functionalized BN was added to the functionalized magnetite suspension during mixing. After stirring for 4 h, a stable suspension containing BN–Fe_3_O_4_ hybrid was obtained.

Table 1 shows the mixing ratios of the cement composite samples containing BN–Fe_3_O_4_ nanostructure. At least 5 samples were fabricated tested for each variable parameter. For all samples, the water/cement weight ratio (w/c) was 0.5. The ratio of 0.5 is a basis and common value for making cement and concretes. In larger than this value, the amount of porosity left within the cement after hydration may increase, and in smaller than 0.5, the performance of the cement may decrease. Fe_3_O_4_ content was kept at a constant value of 0.5% of the cement weight while the weight ratio of BN/cement was 0.025–0.075%. The cement paste was prepared using a high-speed shear mixer (TAT-2500). The cement paste was mixed at 2000 rpm for 5 min.

**Table 1:** Cement composite samples containing various amounts of BN–Fe_3_O_4_ nanostructures.

BN	Fe_3_O_4_	Water	Cement	Reaction nanoparticles together	Specimen
0.00	0.0	50	100	–	C
0.025	0.5	50	100	Nanostructure	BN–F25
0.05	0.5	50	100	Nanostructure	BN–F5
0.075	0.5	50	100	Nanostructure	BN–F75
0.05	0.5	50	100	Separate	BN/F5

The compressive strength test was measured on the 15 × 15 × 15 mm specimens in accordance with ASTM C109/C109 M-11b standard with a displacement rate of 2 mm/min. Flexural strength tests were performed on the samples with dimensions of 10 × 10 × 50 mm with a displacement rate of 0.2 mm/min using the ASTM C78/C78 M-10 standard. Electrical resistivity values of the cement-based samples were performed using the simple two-electrode method. A concrete resistance gauge (RCON, Giatec) was used to record the impedance between two copper electrodes.

A simultaneous thermal analysis (STA) instrument with simultaneous thermogravimetric analysis (TGA) and differential thermal analysis (DTA) was used to quantitatively estimate the thermal decomposition of the hydrate phase. The samples were heated from room temperature (~ 23 °C) up to 1000 °C at a rate of 10 °C per minute under nitrogen flow. The samples were in powdered form for STA measurement.

For measuring the thermal conductivity, the laser flash method was used. In this method, a pulsed laser and a data acquisition board, and Labtech software were used. The specimen was in the form of a disc with a diameter of 13 mm and a thickness of 2 mm.

X-ray fluorescence (XRF) was utilized to investigate the chemical composition of cementitious materials.

X-ray diffraction (XRD) with CuKα radiation at (λ = 1.5406 Å) was employed to determine phase compounds in the materials. All tests were carried out at ambient temperature. In this work, ‘X’Pert HighScore Plus software was used to analyse different peaks.

A JEOL 7001F FEG scanning electron microscopy (SEM) with energy dispersive X-ray spectroscopy (EDX) was used for evaluation of the fracture surface of the cementitious blocks. The SEM was operated in a high vacuum mode at an accelerating voltage of 5 to 15 kV. Transmission electron microscope (TEM) was also employed to characterize the morphologies of the BN and BN–Fe_3_O_4_ nanocomposite.

## Results

### The chemical composition of ordinary portland cement (OPC)

Table 2 shows the chemical composition of OPC measured using X-ray fluorescence (XRF) analysis.

**Table 2:** Chemical composition (wt%) of the OPC used in this work.

Phase	CaO	SiO_2_	Al_2_O_3_	Fe_2_O_3_	MgO	K_2_O	Na_2_O	SO_3_	LOI
Percentage	62.7	20.1	5.4	3.2	1.3	0.5	0.2	2.9	3.5

### Properties of nanostructures obtained

**Figure 2:**
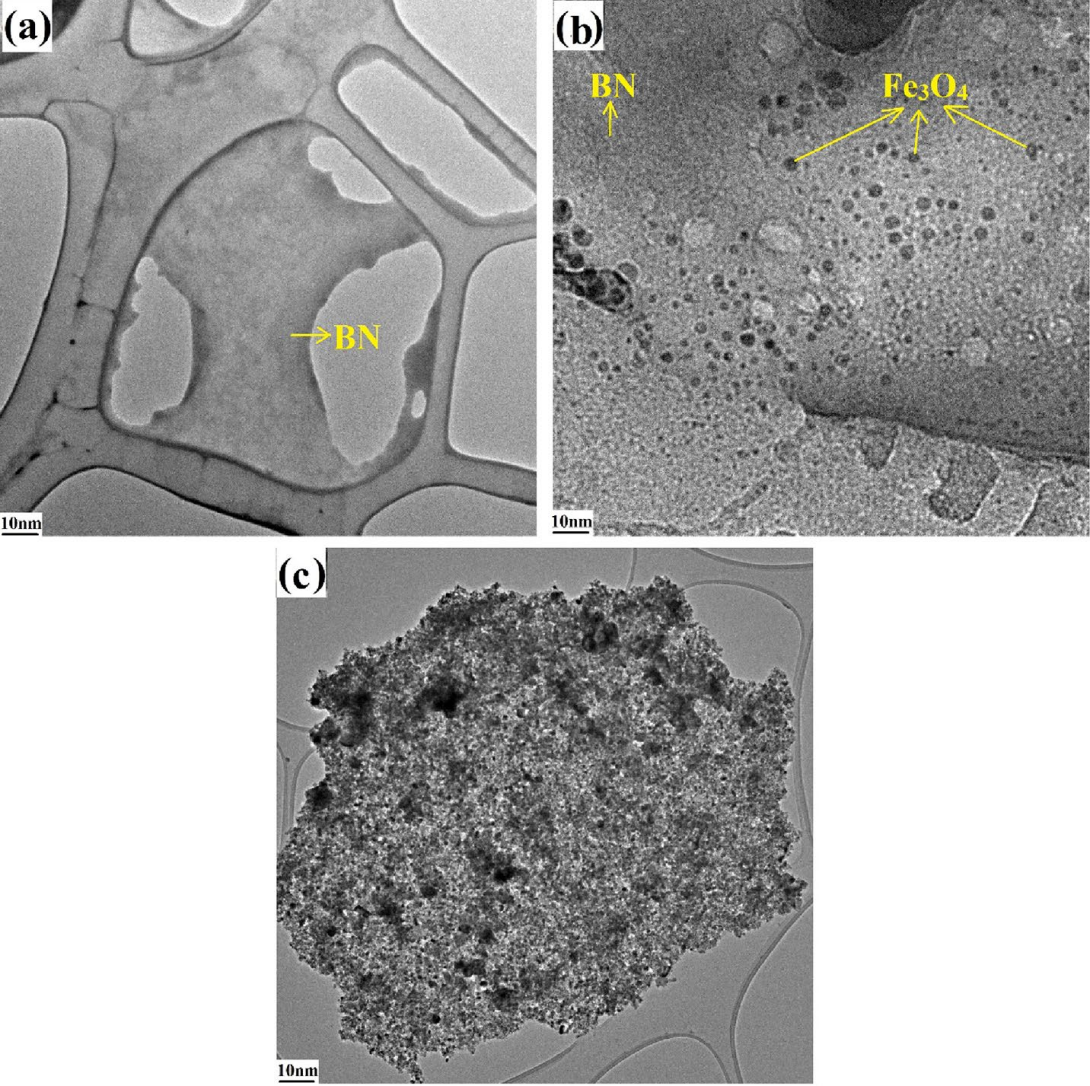
TEM images of the (**a**) BN, (**b**) appropriate BN–Fe_3_O_4_ nanostructure and (**c**) inappropriate BN–Fe_3_O_4_ nanostructure.

As shown in Fig. 2a, after 6 h ultrasonic (200 w), pure BNs have a high aspect ratio and a complete plate structure. BN thickness is monolayer and length up to several micrometers. These morphological characteristics make BN suitable for composite applications.

The morphology and structure of the synthesized BN–F hybrid nanofillers were investigated using TEM. It is known that BN nanosheets form hexagonal planes with smooth surfaces. Figure 2b clearly shows the highly uniform deposition of nano-sized Fe_3_O_4_ on the BN surface. This demonstrates the successful bonding of Fe_3_O_4_ nanoparticles and BN nanosheets together and the formation of BN–F nanocomposites. Figure 2a shows the TEM image of the BN nanosheet. In Fig. 2b, the TEM image of the nanostructured BN–Fe_3_O_4_ is illustrated where Fe_3_O_4_ nanoparticles were added to the BN nanosheet at appropriate intervals and BN and Fe_3_O_4_ could be easily distinguished (sample BN–F5). However, in Fig. 2c, which is related to the BN–F25 sample, more Fe_3_O_4_ nanoparticles were incorporated into BN nanosheet compared to the BN–F5 sample, as a result, Fe_3_O_4_ nanoparticles covered the entire BN surface and were agglomerate, and distinguish between BN nanosheet and Fe_3_O_4_ nanoparticles is difficult. The nanostructure of BN–F nanosheets includes unique features of each Fe_3_O_4_ and BN nanoparticles as well as new hybrid surface roughness enhancement. However, if the Fe_3_O_4_ nanoparticles are added too much to the BN, the number of Fe_3_O_4_ nanoparticles on the BN surface is increased and since the BN nanoplates are very thin they cannot react with the cement particles (Fig. 2c). It is worth mention that the mechanical functions were dependent on the pore structure as well as the “bridge effect” of BN^22–26^. Therefore, the compressive strength was less affected by the addition of nanoparticles at an early age, whereas at higher ages by a natural increase in hydration and consequently reduced cement cavities, addition of nanoparticles had a more noticeable effect on mechanical strength. It was concluded that incorporating a certain amount of each Fe_3_O_4_ nanoparticle and BN nanoplates in BN–Fe_3_O_4_ nanostructure is an excellent choice for reinforcing cementitious materials in terms of compressive strength.

### Mechanical properties

**Figure 3:**
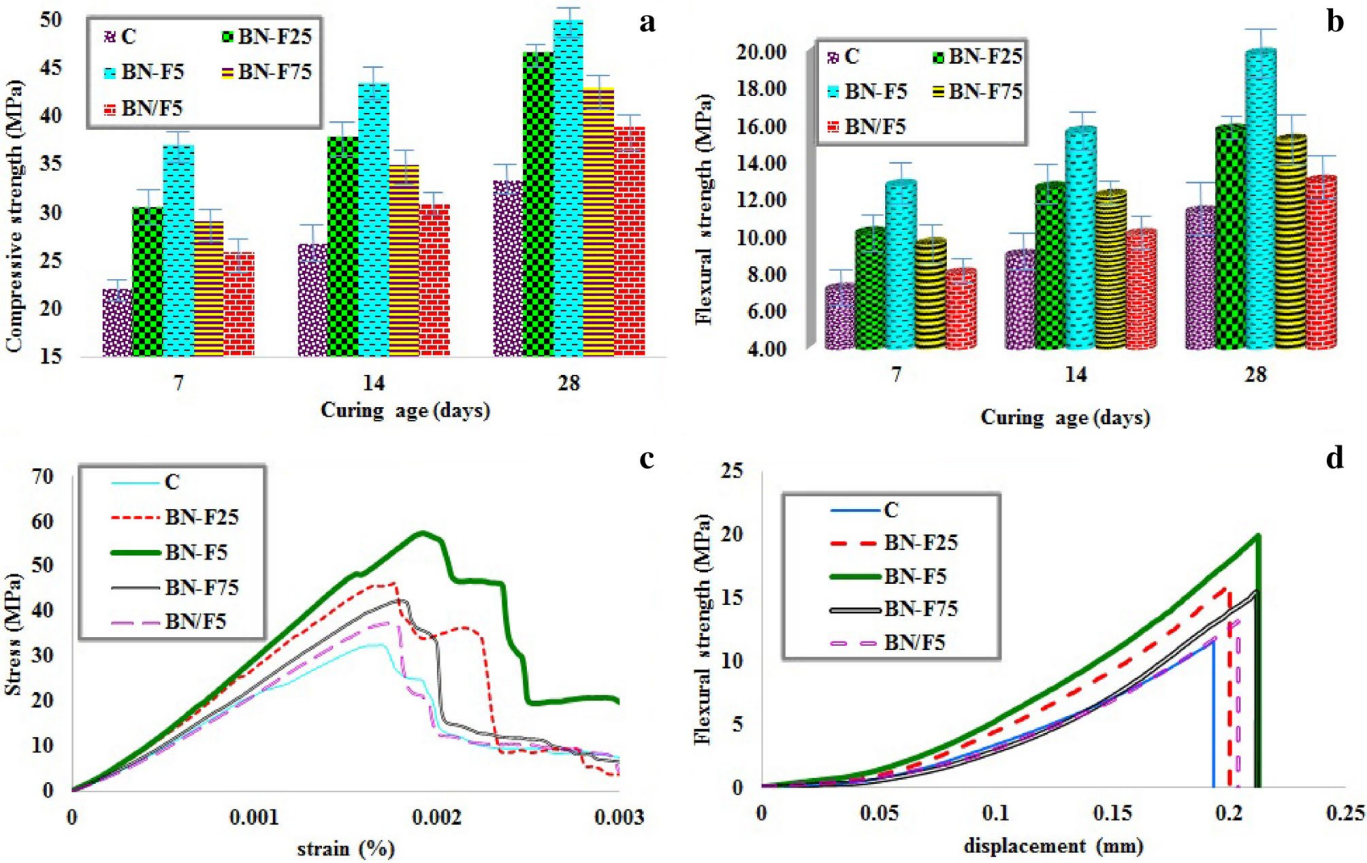
Compressive strength (**a**) and flexural strength (**b**) at different ages of hydration, compressive stress–strain diagrams (**c**) and flexural stress-displacement diagram (**d**) at 28 days of hydration of the cement paste samples containing different amounts of BN–Fe_3_O_4_ nanostructure.

Boron nitride 2D nanomaterial is the stiffest material that has high intrinsic strength and when it is used as fillers, a composite with higher mechanical properties can be fabricated^26^. The corresponding compressive strength and stress–strain, as well as flexural strength and flexural-displacement stress diagrams of the composites, are shown in Fig. 3a–d, respectively. Proper bonding of the nanoparticles facilitates good dispersion of the fillers and assists in their role as the reinforcements in the composite. In addition to the prominent features of each of the Fe_3_O_4_ and BN nanoparticles, their excellent performance is mainly attributed to the excellent bond between the fillers. Furthermore, interaction at the matrix/filler interface due to the hybrid effects of the fillers increases the effective mechanical load transfer from the matrix to the filler and increases the strength of the composites^27^. Proper distribution of Fe_3_O_4_ nanoparticles on the BN surfaces prevents agglomeration. As a result, good dispersion of BN–F is observed in the cement environment. Studies have also shown that the growth of nanoparticles on RGO sheets can also effectively prevent the nanoparticles from the agglomeration^28^.

### Compressive and flexural strengths

The results in Fig. 3 show that the cement containing BN–F has higher mechanical properties than that of neat cement in all ages. The highest increase in compressive strength (67%) and flexural strength (77%) was obtained in 28 days for the BN–F sample. Sample BN–F shows compressive strength and flexural strength of 62–67% and 73–77% of neat cement, respectively, while increasing the compressive and flexural strength of BN/F showed the lowest values among the cement-based composites. This indicates that the bonding and hybridization of the nanoparticles effectively improve the mechanical properties of the cement composite. Even at low values, due to better interaction of the nanoparticles with the matrix phase, a significant increase in mechanical strength is obtained. On the other hand, the dimensions of the individual nanoparticles are likely to be too small to cause a significant deviation of the crack propagation in the matrix. So it is possible that the bonding of these nanoparticles should effectively change the crack propagation path and consequently, it leads to an increase in the fracture toughness of the composite^29,30^. SEM studies in the next section provide evidence for these phenomena.

The results in Fig. 3b illustrate that the addition of nanoparticles significantly improves the flexural load of the cement, which is attributed to the interfacial interaction between the nanoparticles and the cement matrix. The load and flexural displacement values of the cementitious composite containing BN–F are higher than that of BN/F. It indicates that as the dispersion improves, a hard intermediate layer is formed between the cementitious matrix and the BN–F nanostructure that makes the cement deformation harder.

### Stress–strain diagram

The effects of functionalization on compressive strength (maximum stress in the stress–strain diagram) and Young’s modulus (the slope of the stress − strain diagram in the linear region) are investigated. Figure 3c shows the stress–strain diagram of neat cement and cement-based nanocomposites with constant content of nanoparticles. As can be seen, the slope of the stress–strain diagram increased with the addition of nanoparticles, however, for the cement sample of BN/F, the slope of the diagram did not change significantly due to the poor interaction of the nanoparticles with the matrix. On the other hand, the BN–F specimens had a significant effect on the area under the stress–strain diagram due to preventing the crack growth. Finally, it is worth noting how these new nanostructures can improve the mechanical strength of cementitious materials. Since Young’s modulus of the nanostructures is much higher than that of cement, these nanoparticles can certainly be used as reinforcements where stretching is applied^31^. Additionally, previous studies reported that the inclusion of nanomaterials can increase the relative amount of high-density CSH in the cement matrix, which can significantly increase the Young’s modulus of the composites^32^. These results show that the performance of nanoparticles (due to dispersion, size, bonding, and surface roughness caused by nanoparticles hybrid) significantly improved the stress–strain behavior of cementitious composites. The improvement of Young's modulus shows that by improving the interaction of the nanoparticles with the matrix, a hard intermediate layer between the nanoparticles and the cement and causes to restrain the cement deformation.

### Flexural stress–displacement diagram

Figure 3d illustrates the stress-displacement response of the neat cement and their nanocomposites with the same content (0.05 wt%) of BN/F mixture and BN–F hybrids under flexural loading. By introducing a small amount of these reinforcements, the mechanical behavior of the different nanocomposites apparently shows a different enhancing trend compared to the neat cement.

When comparing the mechanical properties of BN/F and BN–F cementitious nanocomposites, it is clear that BN–F exhibits a better reinforcing effect than BN/F. This is attributed to the fact that the improved interfacial interaction, dispersion, and compatibility of BN–F in cement matrix are attained, which are important factors to the mechanical properties of cementitious composites after hybridization^33–36^. In the aspect of stress-displacement responses, BN–F performs better than cementitious nanocomposites reinforced by separate BN/F nanofillers and this demonstrates the synergistic effect of BN–F on the improvement of the flexural properties of cementitious nanocomposites. For example, BN–F achieves the highest flexural strength, elastic modulus, and elongation at break compared to neat cement. BN–F mechanical properties are even higher than those of cement nanocomposites reinforced by BN/F filler. This synergistic effect can be explained by the following two aspects. Firstly, BN–F overcomes the dispersion problem faced by the individual nanoparticles and forming the filler network structure with uniform filler dispersion in the cement matrix. This makes the BN–F transfer the load uniformly in the matrix. Secondly, these hybrid structures have many advantages over traditional nanofillers: (a) The interfacial interaction between BN–F and the cement matrix may be improved by increasing the surface roughness due to Fe_3_O_4_ nanoparticles bonded to BN, which can create more friction and thus prevent the pull-out phenomenon. (b) BN–F hybrids can produce stronger interfacial interactions with the cement matrix than the individual nanofillers. Improving the compatibility and synergistic effects of nanoparticle hybrids on nanocomposites can be seen in previous works^37–39^. (c) besides, the lack of surface functional groups on Fe_3_O_4_ and BN limits both interfacial bond and load transfer in the cement matrix. Thus, by creating functional groups to BN–F, would expect that the interfacial interaction of BN–F with the cementitious matrix would be better than that of BN/F.

### Thermal conductivity

**Figure 4:**
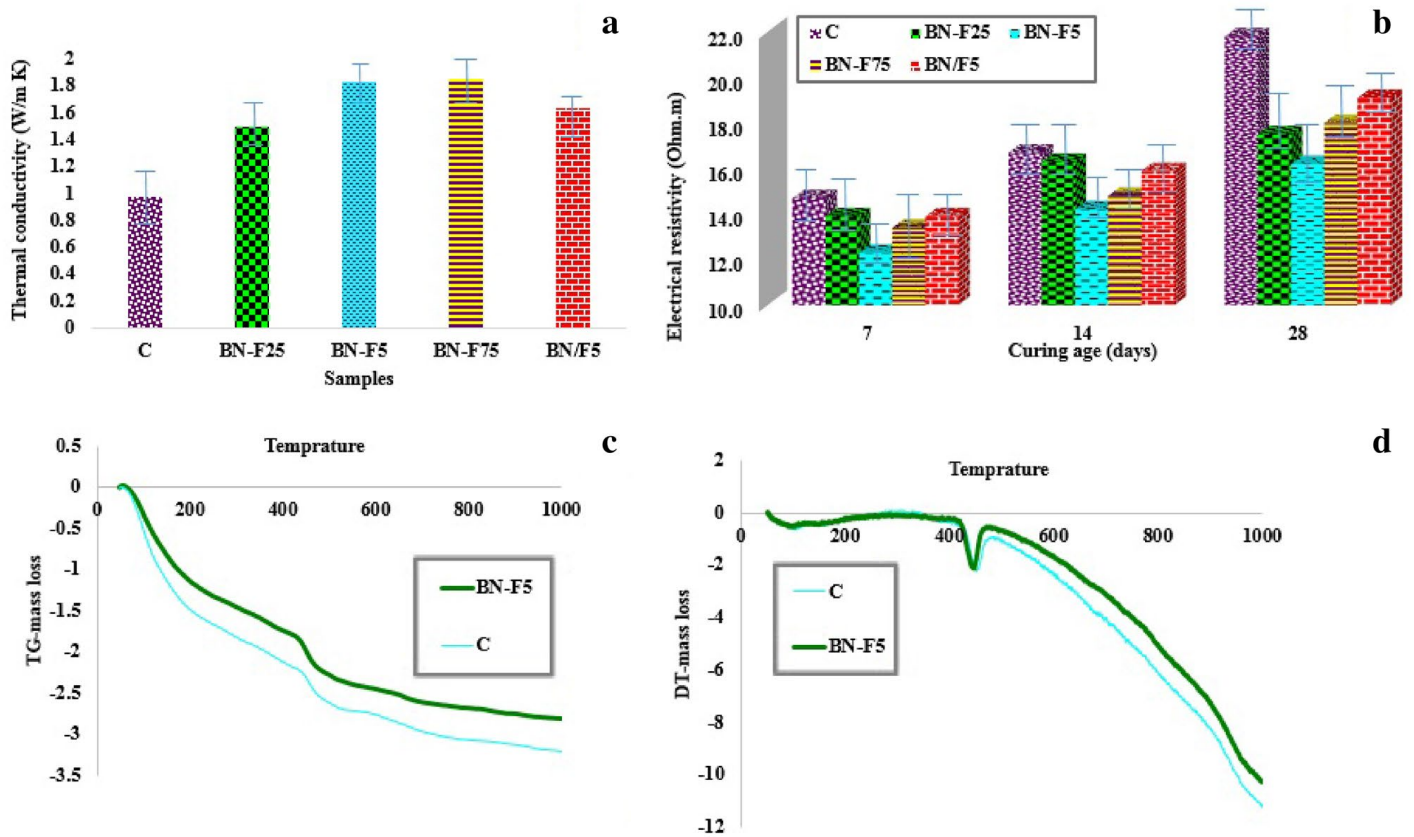
Thermal conductivity at 28 days of hydration (**a**), electrical resistivity at different ages of hydration (**b**), TGA/DTA results (**c**, **d**) at 28 days of hydration of the cement paste samples containing different amounts of BN–Fe_3_O_4_ nanostructure.

The thermal conductivity of the cement composite prepared from BN and Fe_3_O_4_ nanoparticles is plotted in Fig. 4a. Obviously, with the addition of BN nanoplates, the thermal conductivity of the cementitious composite increased significantly. The addition of BN–F nanostructures can provide a higher efficient thermal conductivity network. Also, due to the excellent dispersed structure of BN–F as well as the appropriate surface contact between BN and F (due to the uniform size of Fe_3_O_4_ nanoparticles and their uniform distribution on the surface of BN) the thermal resistance between the fillers reduced, and thus the thermal conductivity is remarkably improved. In general, as the dispersion improves, the mean free path of the phonons also improves, which reduces the energy waste along the heat flux path. As a result, heat transfer efficiency increases.

The thermal conductivity of the pure cement is about 1 Wm^−1^ K^−1^. Cement composites containing nanoparticles show a considerable increase in thermal conductivity in comparison with pure cement. It should be noted that the thermal conductivity improvement in cement-BN/F nanocomposite is not significant. The highest measured thermal conductivity is 1.85 Wm^−1^ K^−1^ at 0.075 w.t% BN–F, which is 91% higher than pure cement. This means that due to the improvement of dispersion of BN–F nanostructures in the matrix, higher efficient heat transfer pathways have been formed in the composite. It is believed that at higher content of BN–F, the thermal conductivity of cement-based composite will raise.

The thermal conductivity of all cement composites with the same filler concentration was tested. Also, the thermal conductivity of neat cement and cement-BN/F (Fe_3_O_4_ and BN separately) composites were used to compare the influence of the surface modification on thermal conductivity. This is because dispersed particles can easily provide heat transfer paths that will facilitate the transportation of the phonon thereby enhances thermal conductivity. On the other hand, due to the relatively low thermal conductivity of Fe_3_O_4_ nanoparticles (95 W/mK) as well as the interfacial thermal resistance or Kapitza resistance occurring at the interface between two materials^40^, the BN–F nanostructure is expected to decrease the thermal conductivity. But according to the constant concentration of the fillers and also improved dispersion of BN–F, thermal conductivity has shown a significant enhancement. On the other hand, cement samples containing nanoparticles separated by both Fe_3_O_4_ and BN (BN/F) had very little effect on thermal conductivity due to the agglomeration of the nanoparticles.

### Electrical resistivity

The electrical resistivity of neat cement is about 15–21 (ohm m) (see Fig. 4b). In longer curing duration, due to fabrication of more hydration products, the microstructure became denser, consequently, the electrical resistivity raised. The presence of BN–F introduces charge carriers into the system, and the charge delocalization on the cement matrix helps to increase the electrical conductivity of the composite. As dispersion improves, the filler particles are enough close to allow electrons to hop across the gaps between them. Increasing the concentration and reaching the threshold percolation concentration can lead to a significant increase in the electrical conductivity or a significant decrease in the electrical resistivity. At this concentration, the conductive filler makes a consecutive conductive network through which electrons can freely travel. It was found that the total electrical resistivity decreases with increasing BN–F content. Fe_3_O_4_ with high electrical conductivity and high surface area are considered conductive additives for composites and can provide the percolation phenomenon at low concentrations^41^. Also, the electrical resistivity of the cement-based composites is influenced by the concentration of the solid hydration products and ionic diffusions through water-filled pores of specimens^42,43^. Since the sample BN–F5 was shown to have the lowest pore concentration and to be the most compact sample, it is reasonable for this sample to exhibit the lowest conductivity/ highest resistivity; the higher experimental values may be attributed to the formation of a percolation network among the BN–F fillers. The percolation network is a system where the nanoparticles are in physical contact, forming a network in the matrix. As is evident from Fig. 4b, the electrical conductivity increases rapidly with an increase in the BN–F nanostructure content up to 0.05 wt% due to the formation of the electrical conduction path in the matrix. Thereby confirming the formation of a percolation network among the BN–F filler particles. the BN–F fillers came in contact with each other when the percolation network among them was formed, leading to an increase in the electrical conductivity of the composite.

The electrical resistivity of the sample BN–F75 increased again, suggesting that 0.05 wt% is the optimum content of the BN beyond which their agglomeration can lower the resistivity due to non-uniform dispersion of the conductive pathways as well as increasing porosity of the sample. This behavior of the BN–F-loaded cement composites is clearly distinct from that of commercial BN/F -loaded composites. The randomly dispersed BN nanosheets and Fe_3_O_4_ nanoparticle (BN/F5) do not tend to form network structure like that in the BN–F5 nanostructure^44–46^. This behavior is attributed to the formation of a percolation network among the BN/F5 aggregate filler nanostructure in the cement paste matrix.

The diagram of the relationship between electrical resistance and conductive filler concentration can be divided into three regions: insulation, semiconductor, and conductive regions^47^. Regardless of the content of nanoparticles, cement composites containing BN/F act as insulators, while BN and Fe_3_O_4_ nanoparticles were properly bonded together, the BN–F cementitious composite became from insulation to a semi-conductor due to improved dispersion of reinforcements.

The comparative data demonstrate that the bonding of the nanoparticles together helps to form a strong interfacial bonding between the matrix and the nanoparticles, resulting in the creation of an effective electrical conductivity network with the cement matrix. Besides, the extended structure of the hybrid filler may also create networks that improve contact regions, result in increasing electrical conductivity of cement-based nanocomposites.

### TGA/DTA analysis

Figure 4c,d shows the TGA / DTA results of the neat cement paste and BN–F at 28 days. In Fig. 4c, the TGA results are plotted to identify the weight loss of cement hydration products and DTA to identify the decompositions of hydrated and carbonated phases. As shown in the Fig. 4c,d, the samples have three main weight reductions. The first major mass loss is dehydration of the C–S–H gels and the hydrated aluminate phases in the range of 100–200 °C. The second major mass loss was around 500° C and is attributed to the decomposition of calcium hydroxide produced by calcium silicate phase hydration of the cement^48,49^. The amount of non-evaporated water content is considered as a weight-loss per gram of cement paste between 105 and 1000 °C as an experimental measurement of the degree of hydration^50^. Figure 4c,d demonstrate that during STA, generally, the diagram of BN–F5 was higher than that of neat cement. It shows that the main weight reduction of BN–F5 was lower than that of neat cement. It means the amount of non-evaporable water in BN–F5 was consistently at a higher proportion than that in neat cement, which is taken as evidence about the role of BN–F in accelerating cement hydration. Besides, the degree of cement hydration was improved with the incorporation of BN–F, illustrating a higher non-evaporable water content.

### The phase structure of cement-based composite

**Figure 5:**
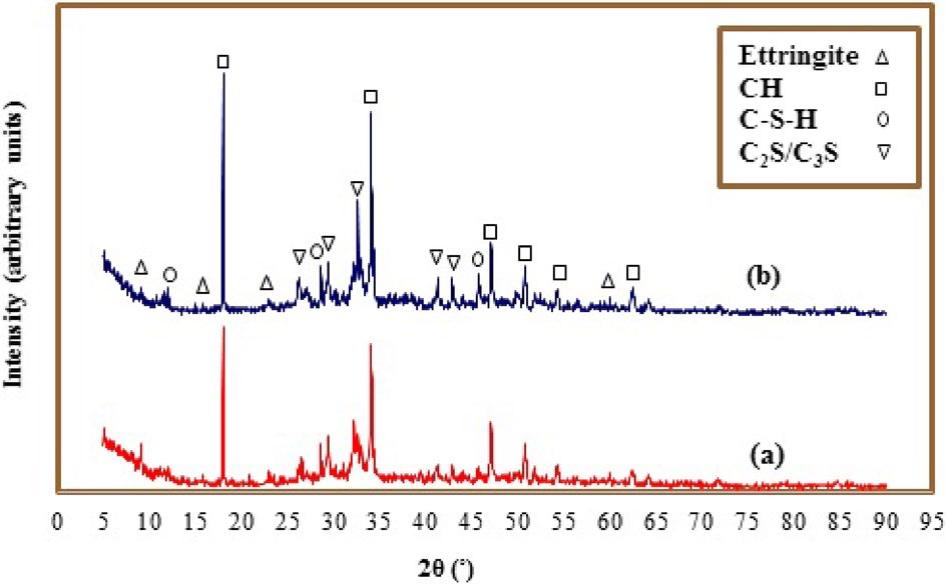
The XRD patterns of (**a**) neat cement and (**b**) the cementitious composite loaded with BN–Fe_3_O_4_ nanocomposite (BN–F5) at 28 days of hydration.

The XRD patterns of simple cement paste and BN–F5 sample with the best mechanical response and porosity after 28 days of processing are shown in Fig. 5. It is clear that a new peak is not visible in the template, but only the intensity of the peaks has changed. Moreover, the addition of nanostructured BN–Fe_3_O_4_ to cement paste has increased the peak intensity of calcium hydroxide (CH) and C–S–H gel^51–53^. It can be seen that the intensities of the peaks corresponding to three major cement hydration phases (CH and C–S–H) increased by adding BN–Fe_3_O_4_ to the cement, which can be attributed to the acceleration of hydration process and the role of nucleation seeding of Fe_3_O_4_ and BN nanoparticles, creating a denser and strong structure. The calcium-silicate-hydrate gel (C–S–H) is an amorphous cement phase, thus its detection through the XRD spectrum is limited^54,55^. The cement paste structure at the nanoscale is affected by the calcium silicate hydrate phase (C–S–H). Basic properties such as strength, breaking behavior, shrinkage and durability, are essentially controlled by the characteristics of C–S–H and porosity^22,56^. On the other hand, with the addition of nanostructured BN–Fe_3_O_4_, the intensity of the peaks related to ettringite has decreased, in other words, the hydration process has been improved. The reason for this phenomenon can be stated that the increase in the phases of CH and C–S–H prevented the growth of the needle-shaped ettringite crystals.

### Microstructure

**Figure 6:**
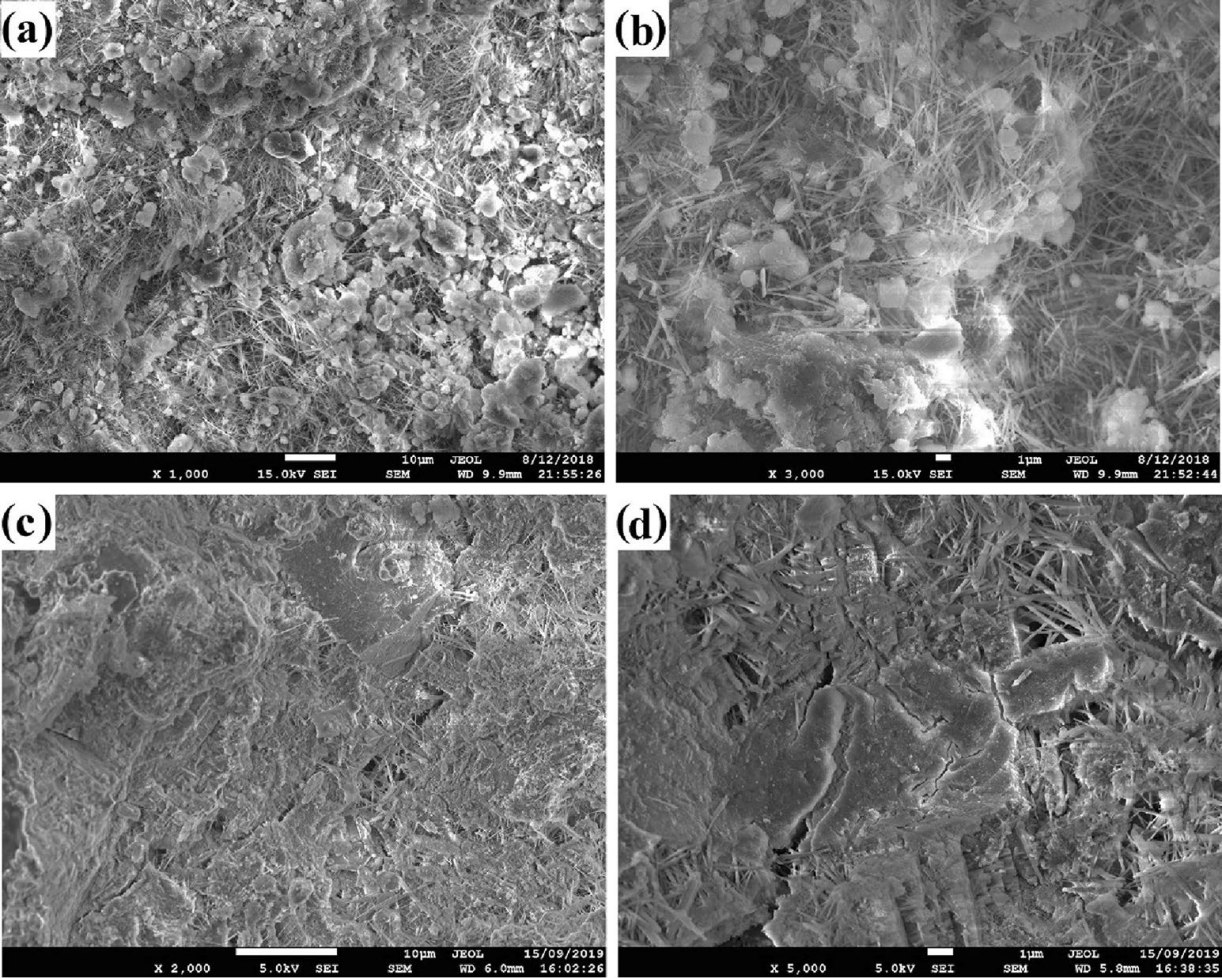
SEM micrographs from fracture surfaces of the (**a**, **b**) neat cement and (**c**, **d**) BN–F5 sample hydrated for 28 days.

The enhanced mechanical strength of cement nanocomposites is attributed to the microcracking by the protruding Fe_3_O_4_ nanoparticles on the surface of BN nanosheets, this can limit the propagation of cracks into large cracks and would further reinforce the cement nanocomposites. Also, protruding Fe_3_O_4_ nanoparticles can cause nucleation in the cavities^57^, which could cause further BN interaction with the matrix and limit crack propagation. The SEM images of the fracture surfaces of neat cement and cement nanocomposites are shown in Fig. 6. It can be observed clearly from Fig. 6a,b that the structural deformation of the neat cement is a brittle failure. However, cement nanocomposites exhibit a united fracture surface, this can be attributed to the matrix shear yielding or cement deformation between the nanofillers. Moreover, the micro-cracks (Fig. 6d) were stopped by reaching the BN nanosheets (covered with a layer of cement), this indicates that BN also plays a role in improving the strength of cement nanocomposites by relieving the stress state. Meanwhile, a high number of micro-cracks (with smaller length) is observed in the BN–F sample (BN with Fe_3_O_4_ protruding nanoparticles). It shows that Fe_3_O_4_ protruding nanoparticles can also transfer stress loaded onto BN, enhance the strength of cement nanocomposites.

SEM, EDX studies were conducted to determine the morphological characteristics and bonding of nanoparticles with OPC hydration products. Interaction of nanoparticles with cement hydration products is related to the nature of the pore-filling mechanism at hydration mechanism^58,59^.

**Figure 7:**
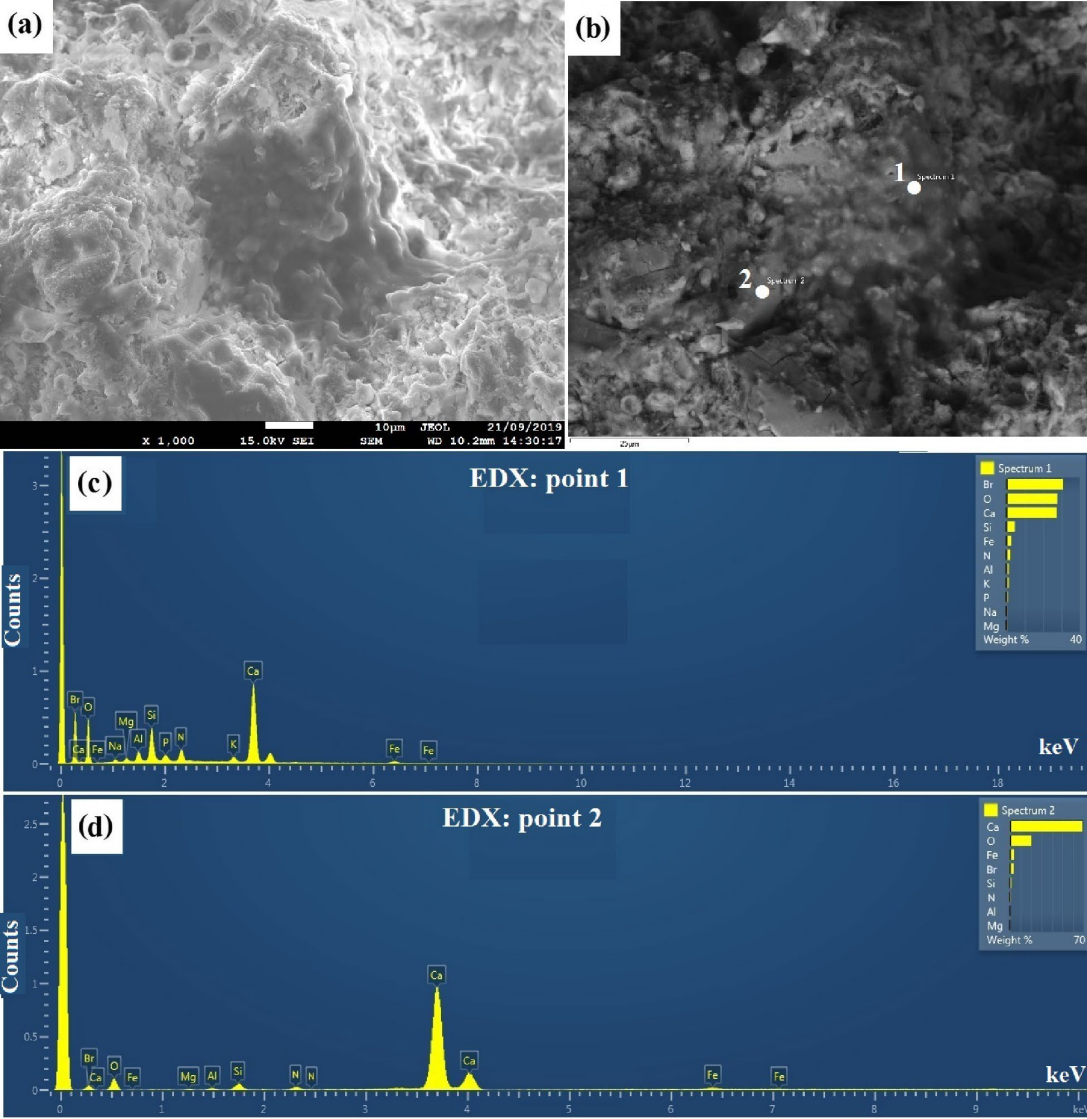
The SEM micrographs (**a**, **b**) and EDX spectra (**c**, **d**) from fracture surfaces of the BN–F5 sample after curing for 28 days.

Figure 7a,b presents the SEM images of sample BN–F5, a different morphology is observed compared to the neat cement sample. As apparent from the images, a thin layer of BN-F can observe in the sediment matrix. As for point 1 (Fig. 7c), high content of Br, N, and Fe element was detected, which illustrate that the lamina in this area are probably BN-F.

According to the EDX spectra, see from the element composition of points 1 and 2 (Fig. 7c,d), high content of Ca, O, Si, element demonstrated the lamina might be the composite of Cement hydration products. This implies that the original BN-Fs are covered by the hydration products, resulting in a stronger interaction between BN-F filler and cement matrix. The EDX results show that the lamina contained a large amount of Br, N, Fe, and calcium, confirming the cross-linking interactions between BN-F nanostructure and calcium ions. The dispersion of this poly-system showed good homogeneity and uniformity, which in good agreement with mechanical strength results. The BN–F5 stabilized sediment sample represented excellent mechanical strength by means of the adhering effect of the poly-system which would closely bond sediment nanoparticles.

### Effect of BN–Fe_3_O_4_ on the restriction of crack propagation

According to the observed failure mechanism of the various nano-fillers, the failure mechanisms of individual fillers and the hybrids in an ideal dispersion condition are further revealed. When the crack front encounters BN, fracture contributes to the major failure mechanisms that may depend on the interfacial adhesion property as well as the length of the BN (pulling and bridging). For BN, the front of the crack is first pinned to the BN, and branches then continue propagating. It is noteworthy that the height level of the bifurcated cracks may be different, which makes the crack path exhausting and dissipate much energy, leading to improve mechanical properties of cementitious composites (similar results have been found by researchers)^60–64^. For BN–F hybrids, there are more complicated mechanisms involving the combination of fracture, crack bridging, bifurcation/deflection, and increased surface roughness than individual nanofillers. In the nanofiller hybrid network, the bifurcation/deflection mechanism introduced by BN–F renders the crack path torturous, and the number of the crack paths increases. On the other hand, improved dispersion allows cracks to deal with a higher number of nanofillers in different height levels, and thus the fracture and bridging mechanisms are strengthened during crack propagation, resulting in more energy dissipation and improved reinforcing and toughening effects. The above failure mechanisms are all based on the good condition dispersion state of the fillers. However, the poor dispersion condition of the fillers in the cement matrix, such as the agglomeration of the nanofillers in the cement matrix, may increase the stress concentration during loading and then lead to premature failure, which severely affects the load-bearing efficiency^49,65^. Therefore, good dispersion of nanofillers is essential. The hybrids prepared by merging BN and Fe_3_O_4_ attained uniform dispersion in the cement matrix, preventing premature failure due to agglomeration and other undesirable failure mechanisms and maximizing its load-bearing efficiency. So BN–F has excellent reinforcing and toughening effects.

Nanofiller length is also an important factor affecting its failure mechanism. Research has shown the CNTs shorter than the specified amount pulled out of matrix during composite fracture; otherwise, they tend to be a fracture^64,66–68^. On the other hand, it was found that despite the short length of CNTs, modification of the CNT surface effectively improved the interfacial adhesion between the CNT and the matrix, which thereby change the failure mechanism from CNT pull-out to CNT fracture^37^. It is worth noting that the CNT length preservation is important to obtain high mechanical performance composites. But any efficient exfoliation of CNT agglomerates would rely on high energy input or external forces during the composite manufacturing process, which meantime results in the shortening of CNTs^69,70^. Therefore, finding a balance point between better dispersion and sufficient length of nanofiller is essential. According to the expanded structure, improved dispersion, and surface roughness, it is reasonable to conclude that the filler hybrids are effective in improving cement composite structure with good reinforcing and toughening effects.

An accurate understanding of how crack propagation interacts with microstructures plays a vital role in designing microstructure for damage tolerance. Research involving careful examination of the surface microstructure of samples from fractured surfaces provides significant knowledge of crack interactions with microstructures. Mechanisms of toughening and fragility are based on ideas such as bridging, deflection by second-phase particles, as well as chemical modification of the crack tip^62^.

**Figure 8:**
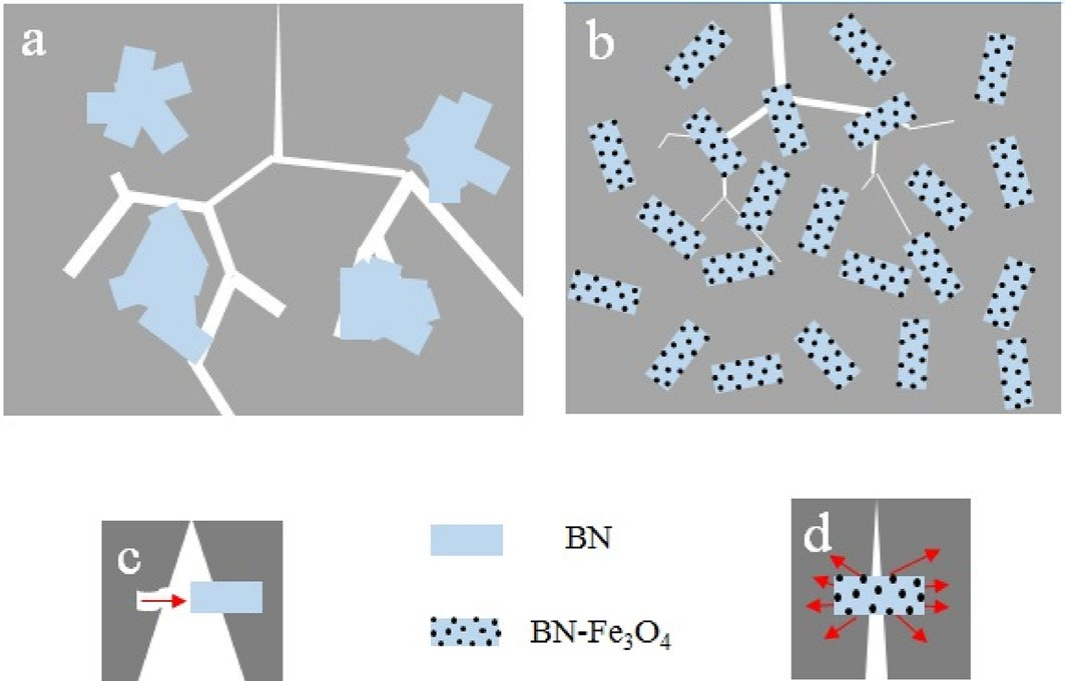
Schematic of crack growth in cement containing BN and BN–Fe_3_O_4_.

Agglomeration affects the BN dispersion in the matrix and reduces the probability of its strike with crack, so the crack can easily grow and propagate in the matrix (Fig. 8a). On the other hand, depending on the orientation of the agglomerated BN nanosheets, there may be several situations concerning the crack. First: the crack moves along the agglomerated BN nanosheets. In this case, since the bonding between the layers is a weak force (van der Waals), sheets are easily separated by crack propagation. The slit spreads between the BN sheets and eventually crosses the layers, resulting in a crack splitting. Second: when the cracks move perpendicular to the agglomerated BN nanosheets, they can be deflected or split into both sides. This provides a zigzag path and a torturous path for moving cracks instead of a straight path. Third: a combination of cracking deviation and separation between BN layers can also occur^60,71^. On the other hand, as shown in Fig. 8b, with the improvement of BN–Fe_3_O_4_ dispersion, the nanostructures are likely to be placed between the cracks, and the activation of crack growth mechanisms is prevented.

As the crack grows, some particles act as a barrier to its propagation. With increasing the applied force, crack growth around particles is easier than passing through the particles (this mechanism is known as the crack bridge). This mechanism works until the matrix is completely fractured around the fiber and the fiber loses its reinforcing effect^72^. Figure 8c,d shows the fracture morphology of BN and BN–Fe_3_O_4_ from their fracture surfaces. The occurrence of the pull-out mechanism is directly related to the particle size and the adhesion between the matrix and the particles. So, not only the particles must have a sufficient length but also a strong adhesion between the matrix and the particles is very effective for stress transfer. Therefore, when the particles have sufficient adhesion to the matrix, the stress transfer is fully developed and the probability of the particles being removed from the matrix becomes weak. However, when the adhesion between the particles and the matrix is insufficient, the stresses transferred to the particles will not be sufficient to prevent the growth of the crack, so the matrix fails in the vicinity of the particles, and the pull-out mechanism happens.

## Conclusions

The simultaneous properties of high electrical and thermal conductivity also with significant improvement in mechanical strength of cement BN–F composite were obtained. Cementitious composites containing BN–F in this study show excellent thermal conductivity, which can have an excellent prospect range of applications. BN–F cementitious composites were prepared using a simple chemical process and electrostatic self-assembly. Adding small amounts of BN–F can be used as a method to control and improve the mechanical and microstructural properties of the composites. Significant improvement in mechanical properties was achieved for BN–F, due to the homogeneous dispersion of Fe_3_O_4_ nanoparticles on the BN surface and the proper binding of Fe_3_O_4_ to BN. As a result, the desired nanostructure in addition to the appropriate length can have good surface adhesion to the cementitious matrix.

Furthermore, TGA results show that the addition of BN–F to the cementitious composite improves the degree of cement hydration. The maximum increase in compressive and flexural strength was obtained at 28 days for BN–F. SEM analysis revealed that BN–F hybrid filler increased the density of cement composite and prevented the growth and propagation of the cracks. These phenomena led to improve mechanical strength and microstructure as well as enhance the degree of cement hydration coupled with good dispersion of BN–F nanostructures as an excellent filler for cementitious composites.
